# Expert Consensus Statement on an Updated Definition of Unintended Weight Loss Among Persons With Human Immunodeficiency Virus in the Modern Treatment Era

**DOI:** 10.1093/cid/ciae407

**Published:** 2024-09-20

**Authors:** Roger Bedimo, David Hardy, Daniel Lee, Frank Palella, David Wohl

**Affiliations:** Department of Internal Medicine, University of Texas Southwestern Medical Center, Dallas, Texas, USA; Division of Infectious Diseases, Keck School of Medicine, University of Southern California, Los Angeles, California, USA; Owen Clinic, University of California San Diego Health, San Diego, California, USA; Infectious Diseases Division, Northwestern University Feinberg School of Medicine, Chicago, Illinois, USA; Division of Infectious Diseases, University of North Carolina at Chapel Hill, Chapel Hill, North Carolina, USA

**Keywords:** HIV-associated wasting, antiretroviral therapy, AIDS, unintentional weight loss, screening

## Abstract

The era of modern antiretroviral therapy (ART) has markedly improved health and survival among persons with human immunodeficiency virus (HIV) (PWH). In the pre-ART era, wasting was associated with HIV disease progression to acquired immunodeficiency syndrome and death. Effective ART has reduced the prevalence and incidence of this pre-ART form of HIV-associated wasting. However, a subgroup of ART-treated virally suppressed PWH continue to lose weight, often accompanied by aging-related comorbidities and/or functional deficits. For this subgroup of patients, the older definition of HIV-associated wasting (HIVAW) cannot and should not be applied. An expert panel comprising the authors of this white paper convened to review the existing definition of HIVAW and to create an updated definition that they termed HIV-associated weight loss, based on clinically defined parameters among contemporary PWH receiving ART. Here, clinical features and laboratory biomarkers associated with HIV-associated weight loss are reviewed and approaches to screening and treatment are considered. Available management approaches, including the use of current US Food and Drug Administration–approved medications for HIVAW and other available therapies are discussed. The expert panel also identified knowledge gaps and provided recommendations for clinicians, payers, and researchers.

Human immunodeficiency virus (HIV)-associated wasting (HIVAW) is a complication of HIV associated with an increased risk for morbidity and mortality [1]. HIVAW is multifaceted and characterized not only by low body weight but also by abnormalities in protein synthesis, proteolysis, and lipid metabolism [1]. The precise pathophysiological mechanisms are not well understood, and their relative contributions vary among affected persons.

In the early years of the HIV/acquired immunodeficiency syndrome (AIDS) epidemic (1981–1996), before the availability of effective antiretroviral therapies (ART), HIVAW was predominantly associated with persistent HIV viremia and advanced immune suppression. Persons with HIV (PWH) commonly presented with progressive moderate to severe body weight loss and body cell mass (BCM) loss as they neared death [2]. HIVAW is an AIDS-defining condition that occurred in nearly one-third of PWH during this pre-ART period [1, 3, 4].

During the late 1990s and early 2000s, the early years of highly effective ART, the prevalence of the severe wasting that was characteristic of advanced AIDS markedly decreased with the increased availability and usage of ART. Nevertheless, weight loss remained a strong predictor of morbidity and mortality even among individuals treated with ART [5]. PWH who experienced a ≥10% weight loss had a 4-fold increased risk of death despite receiving ART [5, 6].

HIV is now a chronic and readily treatable condition because of the availability of effective combination ART that durably controls viral replication [4]. In contrast to the pre-ART era, HIVAW now occurs mostly among virologically suppressed PWH, but the underlying mechanisms, although both related to persistent viral infection, are not exactly the same. Despite effective treatments, a subgroup of PWH receiving modern ART continue to unintentionally lose weight despite virologic suppression. Reports suggested that PWH who experience this type of weight loss experience a greater number of hospitalizations and emergency department visits, have significant declines in quality of life, incur 1.3 times higher healthcare costs [7], and can be 3 times more likely to die than people without such weight loss [8].

Hence, there is an increasing need to raise awareness regarding the continued prevalence and incidence of unintentional weight loss in a subgroup of PWH with controlled viremia [1]. An analysis of the Observational Pharmaco-Epidemiology Research & Analysis cohort in the United States estimated that the prevalence of this type of weight loss among ART-treated PWH was 12% when studied between 2016 and 2020 [9]. The incidence of unintentional weight loss was 7% in the same cohort among PWH without a prior history of wasting or weight loss over a median follow-up of 9 months [9]. Moreover, a claims-based study estimated the prevalence of unintentional weight loss among PWH to be as high as 18% (cumulative prevalence over 2012–2018 was 3.1% annually) [1].

Given its manifesting as predominantly sustained weight loss in the setting of controlled HIV, this contemporary presentation of HIVAW has been redefined as HIV-associated weight loss (HAWL), and awareness of its existence is critical to gain an understanding of its underlying pathogenic mechanisms and risk factors (Figure 1). More current clinical data are needed to update medical guidelines addressing HAWL screening, diagnosis, and management. With these knowledge gaps in mind, an expert panel convened in October 2023 to achieve consensus on a clinically relevant definition of HAWL and to make practical recommendations for its diagnosis and treatment. Specifically, the panel met to achieve the following objectives:

- Redefine HIVAW in the context of the current clinical landscape
- Review laboratory biomarker and other tests used to diagnose HIVAW and HAWL
- Provide best approaches for HAWL screening
- Make practical clinical suggestions for management of HAWL in aging patient populations
- Provide perspectives on currently available treatments, both US Food and Drug Administration (FDA)–approved medications and other commonly used therapies
- Develop recommendations for HAWL treatment.

**Figure 1.**
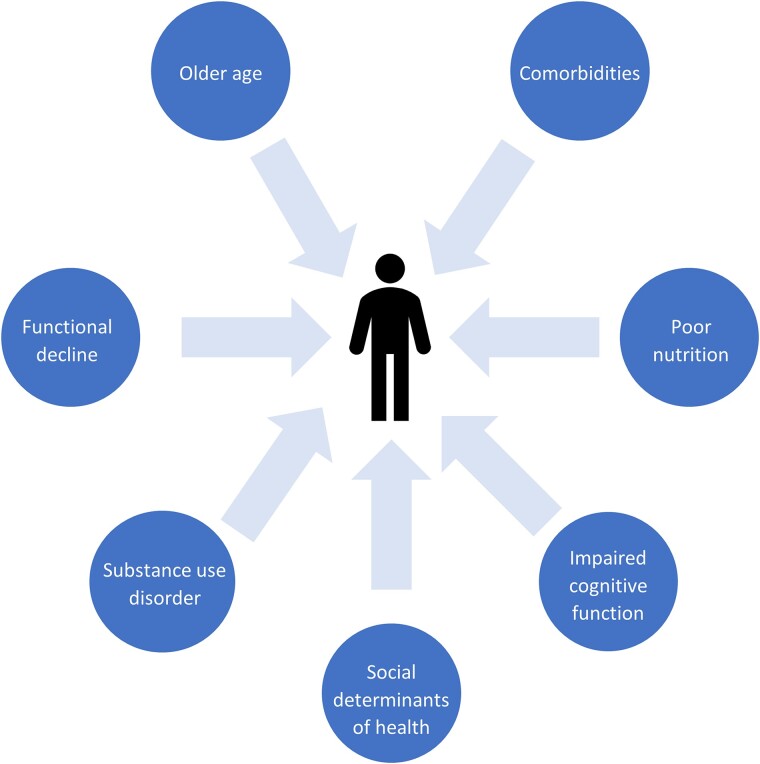
Factors beyond HIV that influence weight loss in a person with HIV.

Following the expert panel meeting, a literature review on each topic discussed was conducted using PubMed or via a keyword search online from 1985 to 2023. Recommendations were summarized from the meeting and were reviewed by each panel member.

The following guidelines and recommendations have been developed to provide awareness and education for clinicians caring for PWH. Other stakeholders, including payers, whose decisions often impact a patient's healthcare medication access, may also benefit from these guidelines and recommendations when evaluating financial coverage of clinical treatment and medical therapies. The convened expert panel offers these recommendations to support improved care for PWH with continuous unintentional weight loss.

## HISTORICAL DEFINITION OF HIVAW

Early in the HIV/AIDS epidemic, and before the modern ART era, HIVAW was widely prevalent among people with an AIDS-defining diagnosis. The Centers for Disease Control (CDC) defined HIVAW as >10% loss of baseline body weight plus either chronic diarrhea or chronic weakness or fever for ≥30 days [10]. This CDC definition was further expanded to include PWH with <90% ideal body weight (or body mass index [BMI] <18.5) and >10% weight loss from premorbid maximum over a 12-month period or >5% weight loss in the previous 6 months [11].

Historically, the CDC definition, which was developed for epidemiological purposes, was easy to apply because weight loss and BMI could readily be measured [12]. The expanded definition included PWH who met relaxed weight loss criteria, although body weight criteria did not take into consideration cultural or sex differences [13] or account for the possible presence of malnutrition, loss of BCM, or from lipoatrophy induced by thymidine analogue nucleoside reverse transcriptase inhibitors [14]. This early phenotype of HIVAW put forth by the CDC largely reflects catabolic processes occurring from uncontrolled HIV viremia; thus, HIVAW was appropriately included in the definition of AIDS-defining illnesses. This clinical presentation is still relevant among PWH who do not receive ART (eg, in geographic locations where effective HIV treatment is not readily available) or among nonelderly, ART-naive PWH.

Despite evolution in the clinical presentation of HIVAW during the modern ART era, the expert panel observed that clinicians, researchers, and the CDC continue to use this historic definition, which underscores a collective impression that HIVAW was an issue of the pre-ART years and is largely no longer relevant. Many recently trained clinicians who treat PWH have learned this traditional definition and continue to associate HIVAW only with PWH who have advanced HIV disease. Of equal importance, concern was expressed that payers may deny prescriptions for effective FDA-approved therapies for HIVAW because affected PWH do not meet the historic CDC definition of HIVAW.

## HIV-ASSOCIATED WEIGHT LOSS

### Rationale for a Modern Definition of HAWL

Recent evidence supports seeking consensus on a singular definition of HIV-associated unintentional weight loss as multiple definitions can cause confusion and mischaracterize certain patient groups [9]. An updated clinically relevant definition of this clinical syndrome in the modern ART era will promote identification of PWH who currently have or are at highest risk for its potentially life-threatening consequences, ensuring that all stakeholders are unified in their approach to diagnosis and treatment. Additionally, a revised definition can facilitate improved assessment of HIV-associated unintentional weight loss epidemiology, as well as its determinants, correlates, and outcomes.

Understanding the diversity among persons at greatest risk of HAWL is imperative because the health consequences of this condition are variable [9]. Broadly, HAWL represents unintentional weight loss that would not be better explained by another concurrent illness or condition beyond HIV infection itself. Unintended weight loss may be associated with any combination of intersecting or overlapping loss of lean body mass (LBM), physical frailty, sarcopenia, muscle wasting, reduced physical functioning, and declines in cognition; with the current population of PWH at greatest risk being primarily older than age 50 years, HAWL may therefore constitute or be a component of geriatric syndromes [15–17]. However, unintentional weight loss can and does occur in younger PWH, often among persons with competing risks for weight loss, but the findings can be more subtle and appear more gradually than with historically defined HIVAW [9]. It may be important to differentiate PWH who are experiencing unintentional weight loss from a comorbidity versus frailty, as treating weight loss caused by these conditions may differ in terms of diagnosis and management. Although modern ART, particularly use of second-generation integrase strand transfer inhibitors, has been associated with weight gain, not all PWH who receive such therapy gain weight [9]. Some PWH who have received effective ART for many years and have no underlying comorbidities may still experience unintentional weight loss from various factors (eg, medication side effects, nutritional deficiencies) [8]. The expert panel agreed that HAWL was more representative of contemporary clinical presentation of unintentional weight loss among PWH than the historical definition of HIVAW.

### An Updated, Clinically Relevant Definition for HIVAW in the Modern Era of ART: HAWL

HAWL is sustained, unintentional weight loss in PWH that:

- Occurs in the absence of a concurrent illness or condition (other than HIV infection) that could readily account for such weight loss,
- Is characterized by >5% loss of premorbid body weight over 6 months or >10% loss of premorbid body weight over 12 months [18] (in the absence of objective of weight loss, low BMI [<20 kg/m^2^] is sufficient) [19],
- May, in some cases, be accompanying reductions in physical functioning, including difficulty with completing activities of daily living, low physical strength, and slow gait speed.

We believe that evaluating PWH using this updated definition will improve detection and diagnostic accuracy of HAWL, thereby allowing for timely initiation of appropriate treatment.

### Recommendations

The expert panel recommends that, although clinical presentations may appear similar, HIVAW and HAWL have distinct underlying causes that differentially characterize a patient's health and treatment needs. Therefore, HIVAW and HAWL should be defined as 2 similar but distinct complications of HIV. As such, the panel proposed differentiating HAWL from the classic HIVAW by whether the PWH under evaluation have suppressed plasma HIV viremia.

- PWH with prolonged nonsuppressed HIV viremia (viral load [VL] >200 copies/mL), weight loss is predominantly secondary to a catabolic process characterized by chronic inflammation and immune dysregulation that can lead to the development of AIDS; the former CDC definition of HIVAW should apply.
- PWH receiving ART with suppressed HIV viremia (<200 copies/mL) and unintentional weight loss should be identified as meeting the contemporary definition of HAWL.

## SCREENING FOR HAWL IN ROUTINE CARE

### Screening for HAWL

Screening for weight loss at each clinic visit is essential for early detection and management of HAWL. To date, the standard for determining the percentage of weight loss is by direct measurement of weight using a calibrated scale and comparison to a prior similarly measured weight. After the updated weight and height measurements have been obtained, BMI can be calculated. These are the 2 required standard objective quantitative measurements that should be used to screen for HAWL.

In the sections that follow, the expert panel weighed in on the use of several additional measurements, diagnostic tests, or biomarkers that may previously have been used to support a diagnosis of HIVAW. Other than serial weight and BMI measurements, no objective indices for routinely performed clinical tests or specific laboratory values have demonstrated use as sensitive or specific markers for HAWL.

### Screening for HAWL by Weight Measurements and BMI

Serial weight measurements and BMI are the easiest and most cost-effective methods for identifying unintentional weight loss among PWH. These measurements have traditionally been documented during routine in-person examinations. However, the coronavirus disease 2019 pandemic ushered in a new era of telehealth medicine, during which documentation of serial anthropometric measures is more challenging. Nevertheless, patient-reported measures of height and weight—which can be aided by the growing use of remote and wearable technologies—can be used to identify unintentional weight loss. Several studies have indicated good to moderate correlation between clinician- and patient-reported measurements, suggesting that self-measurement could be an acceptable alternative method of data collection [20–22]. Conversely, evidence exists suggesting that patient-reported height measurements are overestimated, whereas weight measurements are underestimated [20, 21, 23]. Moreover, payers have mandated evidence of documented weight before certain treatments will be financially covered. Taken together, these data support the assertion that self-reported measures should not replace clinician-measured weight and calculated BMI during in-person clinic visits. Nevertheless, clinicians increasingly recognize the value of educating patients in the undertaking of self-reported serial weight measurements to identify any unhealthy weight changes.

### Additional Screening Measurements for HAWL: Measuring Lean Body Mass

#### Dual-energy x-ray Absorptiometry Scans

Dual-energy x-ray absorptiometry (DXA) is a noninvasive procedure that measures body composition, bone density, and skeletal muscle mass. Previously, DXA was considered the gold standard for measuring fat mass [12], but its reliability for accurate measurement of skeletal muscle mass has been challenged [24]. Because DXA scans are most commonly used to measure bone health and fat mass, some healthcare facilities and payers may view this technique as investigational in evaluating unintentional weight loss among PWH. Therefore, the practical use of DXA to screen for HAWL is limited to healthcare facilities where providers have adequate experience with its performance and interpretation.

#### Bioelectrical Impedance Analyzer

The bioelectrical impedance analyzer (BIA) is an inexpensive and noninvasive instrument that was widely used in measuring BCM during the early 1990s to estimate body composition. LBM and fat mass are estimated indirectly using the resistance of electrical current flow through body tissues; resulting measures are then calculated using an appropriate equation. Because of a lack of standardized software and equations to assess BIA data, large variability in readings has been observed [12, 13]. LBM measurements can be inaccurate depending on a person's hydration status [13]. Even the consistent anatomical placement of electrodes can provide inconsistent measurements. Furthermore, BIA measurements among PWH do not provide any mortality prognostic value compared with longitudinal measurement of weight loss because LBM is not being directly assessed [5]. Many healthcare facilities have moved away from using BIA because of these limitations. However, some payers still require BIA for routine analysis. Given the poor availability and lack of correlative information regarding weight loss associated with using BIA, the expert panel agreed that there is limited value of such measurement in determining loss in LBM to evaluate eligibility among PWH for weight loss treatments compared with serial weight determinations.

#### Nutritional Ultrasonography

One emerging tool for screening skeletal muscle mass and muscle quality is nutritional ultrasonography [25, 26]. Muscle ultrasound is an inexpensive and readily available technique that can be implemented in clinical practice and has a low-to-moderate diagnostic accuracy for diagnosing sarcopenia. Its use in HIV-associated wasting/weight loss has not been investigated.

### Biomarkers

The production of proinflammatory cytokines is believed to play a role in HIV-associated wasting/weight loss [27, 28]. Specifically, tumor necrosis factor α (TNF-α) has long been implicated in AIDS-related illness [27], such as unexplained weight loss. Elevated peripheral blood levels of interleukin 6 (IL-6) have been linked to weight loss and cachexia [29], have been reported among patients with HIVAW compared with healthy controls (*P* < .05) [28], are higher among PWH compared with healthy controls, and in the pre-ART era appeared to increase with disease progression [30]. However, no data are available that identify a distinct correlation between TNF-α levels and the magnitude of weight loss [31] nor has a direct link between IL-6 levels and the development of HIVAW and cachexia been shown [30, 32]. In general, elevated peripheral blood levels of inflammatory cytokines have been identified as catabolic markers among PWH in association with advanced AIDS and may not be as relevant for the ascertainment of wasting among virally suppressed PWH who develop HAWL.

Nonspecific markers of malnutrition, such as serum albumin and prealbumin, have been assessed in PWH [33, 34]. Although easy to measure, low levels of albumin/prealbumin are not specific to wasting but may be caused by concurrent comorbidities (eg, liver, kidney disease). Furthermore, the severity of wasting may have to be substantial for low albumin levels to be present. Other potential serum biomarkers include those associated with sarcopenia (eg, oxidized low-density lipoprotein [35]) or metabolic abnormalities as evidenced by markers such as leptin and adiponectin [36]. However, these markers are not specific for HIVAW or HAWL, and their use not been validated for its diagnosis.

### Measurements of Endurance and Functional Status

Standardized tests that measure endurance and functional status in PWH with or at risk for developing HAWL remain unknown, although there is growing evidence that assessments originally developed for and validated in geriatric populations could be applied to PWH. Precise determinations as to which geriatric assessments are most appropriate for use in PWH are evolving. The advantages and disadvantages of several validated measures [37, 38], including timed walk, grip strength measurement, rise from chair, and questionnaires assessing instrumental activities of daily living (iADLs) are reviewed here, as are patient-reported measures of endurance and functional status.

Timed walk tests are well-known validated assessments that only require space and a stopwatch to measure reduced walking speed and physical endurance, depending on the length of the walk. PWH have slower baseline walk times on the 6-minute test compared with healthy persons of the same age without HIV [39]. The 6-minute or 400-meter walk tests are useful in identifying subtle impairments of endurance, and their successful completion may indicate a high level of physical endurance [37]. A shorter 4-meter walk test may be more practical as it requires less time and space. Although these walk assessments are quick and well validated [37], their use among PWH to date has primarily been in research settings, and their correlation with weight is largely unknown.

A weak grip strength has been associated with increased mortality [37]. Grip strength requires a handheld dynamometer for measurement, and the instrument's regular calibration is required to ensure accuracy. Dynamometer-obtained measurements can be affected by health issues such as arthritis [37]. One study found that PWH who had recovered from wasting showed weaker grip strength compared with PWH without wasting [40].

Chair rise time is another fast and easy way to measure endurance. Chair rise times assess lower limb function and screen for poor locomotor function [39]. Persons are asked to stand from a sitting position (5 or 10 times) within a set period (eg, 30 seconds). As with other physical function measurements, persons with arthritis or severe joint impairment (especially knee issues) may be unable to complete this assessment [37]. PWH with slow chair rise times have been identified as having higher risk for subsequent falls over time [39]. Correlations with weight loss are unknown.

Patient-reported measures can also be useful to evaluate subtle changes in endurance or physical function. With ubiquitous access to smart devices, patients can track exercise tolerance and physical activity using smartphone or smartwatch applications, such as a pedometer or daily step counter. Online questionnaires have also been developed for PWH to help assess whether weight loss and physical functioning may be due to HIVAW or HAWL [41].

Patient-reported questionnaires on iADLs or ADLs are useful tools to assess whether patients experience difficulty or dependency in completing routine tasks [42]. iADLs measure higher level tasks of daily living that are necessary for independent living. These questionnaires evaluate one's ability to perform daily activities such as doing laundry, using the telephone, and preparing food. ADLs measure how difficult it is to complete tasks involving basic care and independence such as bathing, dressing, and eating. A high dependency on other persons, as assessed by iADLs or ADLs, historically has been strongly associated with having an AIDS diagnosis and low CD4^+^ T-cell counts [42]. Collecting subjective patient-reported assessments together with objective routine measures of HIV treatment response (eg, CD4^+^ T-cell counts, HIV VL) and general health (eg, weight, strength, mobility) among PWH can help provide a clearer picture of overall state of health, although correlations with weight loss are not clear.

### Diagnosis of HAWL Using an Updated Screening Algorithm

**Figure 2.**
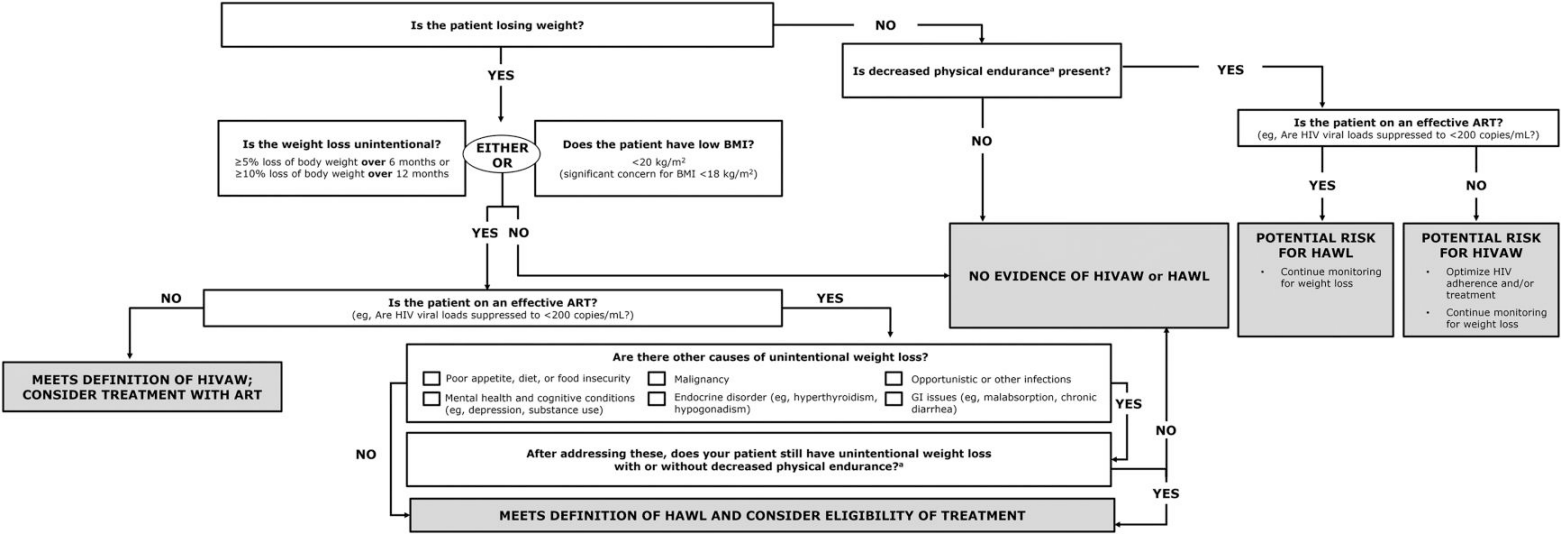
Screening algorithm for HIVAW versus HAWL in PWH in the modern treatment era. ^a^Decreased physical endurance defined as difficulty completing activities such as activities of daily living (ADLs) or instrumental ADLs (iADLs). Abbreviations: HAWL, HIV-associated weight loss; HIVAW, HIV-associated wasting; PWH, people with HIV.

As noted previously, guidelines for diagnosing HIVAW were developed beforehand [12, 13]. However, these older guidelines are inadequate to screen for HAWL because these individuals are usually receiving ART and generally do not experience high-level prolonged HIV viremia. Thus, the following algorithm was developed to assist clinicians in diagnosing HAWL (Figure 2). Clinicians are encouraged to use the algorithm for diagnosing HAWL and to incorporate regular screening into routine clinical care of PWH.

#### Unintentional Weight Loss

PWH must first meet the criteria of unintentional weight loss, defined as ≥5% loss of body weight over 6 months or ≥10% loss of body weight over 12 months compared with premorbid weight [18]. For similar weight loss occurring over shorter periods (eg, over 3 months), acute weight loss may signal an alternate cause that may be unrelated to HAWL, including comorbidities, malignancies, or infections, requiring further evaluation.

For PWH who may be experiencing HAWL and/or are unable to provide documented weight measurements, a BMI of <20 kg/m^2^ for a PWH receiving virally suppressive ART justifies diagnostic evaluation for HAWL. Clinicians should also assess patient functional status, even though this is not a requirement for a HAWL diagnosis. If evidence of unintentional weight loss has been established, further screening for HAWL should be undertaken.

Optimally, in-person clinic visits for routine physical examinations are recommended to record serial weight measurements. Patients who are evaluated with telehealth visits should be educated regarding self-monitoring of weight using a running, recorded log to note any trends in unintentional weight loss and reduction of physical function. Clinicians should document patient-reported weights in the medical record whenever possible.

#### Adherence to ART

A high level of adherence to effective ART documented by continuous HIV suppression (VL <200 copies/mL) is essential for ART treatment success. Nonsuppressed VL (>200 copies/mL) values should prompt repeat measurement and assessment, and reasons for suboptimal virologic suppression, including nonadherence to ART, should be investigated. As mentioned, prolonged high-level viremia might suggest that weight loss could be due to HIVAW, not HAWL.

#### Evaluation for Comorbidities and Other Conditions

Comorbid illnesses that may contribute to unintentional weight loss should be systematically investigated and identified among affected PWH. Eliciting information as to whether observed weight loss is intentional or whether food insecurity exists should be undertaken. This necessarily includes careful questioning regarding dieting, use of appetite suppressants or medications taken specifically for weight reduction, (eg, glucagon-like peptide-1 receptor agonists), and use of recreational substances (eg, stimulants) that may be associated with unintentional weight loss. Of note, the use of glucagon-like peptide-1 agonists can reduce LBM, as well as fat mass and overall weight [43]. Furthermore, reductions in cognitive functioning may result in weight loss or occur because of weight loss, justifying cognitive screening when weight loss is observed [44]. Other comorbidities, including malignancies, depression, endocrine disorders, diabetes, opportunistic or occult infections, and gastrointestinal complaints such as diarrhea and nausea should be screened for and addressed.

#### Management of HAWL

If unintentional weight loss persists (with or without decreased physical endurance) and comorbid conditions or other causes of unintentional weight loss have not been identified, the patient should be regarded as having met the definition of HAWL, and treatment should be considered.

### Recommendations

- Screening for weight loss at each clinic visit is essential for early detection of and intervention for HAWL
- Serial weight and BMI measurements remain the easiest and most cost-effective methods for identifying unintentional weight loss among PWH
- Although no routinely collected laboratory or functional status tests or biomarkers are specific to a diagnosis of HAWL, these tests may provide supportive evidence
- A diagnostic evaluation to rule out comorbidities, medications, and/or recreational drugs associated with unintentional weight loss should be undertaken
- Clinicians should consider using the screening algorithm (Figure 2) to aid in the diagnosis of PWH who have or are at risk for HAWL
- Once a working diagnosis of HAWL is made, treatment for this condition should be considered.

## AGING AND HAWL

As a result of modern ART, PWH are living longer with life expectancies approaching those of the general population [45]. It is estimated that by 2030 nearly three-quarters of PWH will be aged >50 years and one-half will be aged >60 years [46]. As PWH enjoy longer life spans, the achievement of optimal “health span” (duration of time during which an individual maintains optimal overall health and functional status) is critical for aging persons with or without HIV [47]. Healthy aging involves maintaining physical activity, cognitive function, avoidance of chronic systemic illnesses and organ dysfunction, and maintenance of social and psychological well-being to remain independent as long as possible [48].

Advancing age often increases the risk of developing HAWL [9]. PWH may experience an acceleration in physiological aging compared with similarly aged persons without HIV [49]. Possible etiologies associated with this phenomenon include higher systemic levels of chronic inflammation, immune dysregulation, and accelerated cellular senescence. Numerous age-related comorbidities, which generally are geriatric in onset for the general population, may occur among PWH up to 16 years earlier than in persons without HIV [50]. These include metabolic, cardiovascular, neoplastic, and neuropsychiatric conditions [51] as well as increased risk for sarcopenia, weight loss, and reduced functional status [50], which are key risk factors for HAWL. Therefore, to enable a timely diagnosis of HAWL, the care of aging PWH should include proactive vigilance in screening for and addressing aging-related comorbidities, which can cause or lead to unintentional weight loss. Providers should consider that low weight and functional decline may be partially attributable to aging-related non-HIV comorbidities. Accurate ascertainment of causes of unintentional weight loss among PWH will facilitate more prompt initiation of targeted interventions, both pharmacological and nonpharmacological, to mitigate low weight, prevent further functional decline, and improve healthy life spans among PWH.

Compared with persons without HIV, PWH comprise a group burdened with more adverse social determinants of health, including housing and food insecurity, mental illness, substance use disorders, external and internal stigma, and isolation [52]. Older PWH are even more at risk of adverse social determinants of health; these create barriers to the overall acquisition and maintenance of optimal healthcare, including treatment of HAWL. Among the Multicenter AIDS Cohort Study and the Women's Interagency HIV Study cohorts, stigma has been found to be an important predictor of adverse medical outcomes because it influenced the quality of healthcare received [52]. Furthermore, homelessness and housing instability have been associated with reduced ART adherence [53, 54], thereby predisposing PWH to poorer HIV control, which could further lead to disease progression and HIVAW.

Economic stability is another social determinant of health that may negatively affect the level of care that PWH receive. Among participants in the Observational Pharmaco-Epidemiology Research & Analysis cohort, a greater proportion of persons with HIVAW or HAWL were enrolled in Medicaid/Medicare than other types of insurance [9]. It is possible that PWH enrolled in Medicaid/Medicare may have been more vulnerable to HIVAW as a result of lack of access to prevention and treatment services for comorbidities and/or HIV-related care.

It is clear that the development and implementation of measures aimed at reducing the negative impact of adverse social determinants of health is essential to optimize outcomes of PWH, particularly older PWH for whom a convergence of non-HIV competing risks (both medical and social) may increase the risk of HIVAW/HAWL [55, 56].

### Recommendations

- Screening for and treating aging-related non-HIV comorbidities is imperative for older PWH
- Unintended weight loss in older PWH should not be dismissed as merely “due to aging.” Making a timely diagnosis of HAWL can be an important first step in preventing further weight loss and functional decline
- Optimal care of older PWH requires work with other healthcare professionals to ensure that patients have adequate support structures and care access to reduce the impact of adverse social determinants of health
- When confronted with a patient who has a decline in physical function and weight, clinicians should consider both pharmacological and nonpharmacological interventions.

## PHARMACOLOGICAL AND NONPHARMACOLOGICAL INTERVENTIONS, INCLUDING TREATMENT GOALS

### Pharmacological Treatments

Several pharmacological treatments have been used in attempts to mitigate unintentional weight loss among PWH. However, FDA-approved options for the treatment of HIVAW/HAWL are limited (Table 1) [13, 27, 57–73]. Because clinical trials are conducted under widely varying conditions, efficacy and safety observations for drugs should not be directly compared among different studies and may not reflect the rates observed in clinical practice.

**Table 1.** Pharmacological and Nonpharmacological Interventions

Pharmacological Interventions	Intervention	Principal Routes of Administration		Approval Status
Appetite stimulants [59, 60]	Megestrol [59]	Oral suspension		FDA-approved for the treatment of anorexia or unexplained significant weight loss in patients with a diagnosis of AIDS
	Dronabinol [59]	Soft gel capsules (2.5, 5, or 10 mg); oral 5-mg/mL solution [60]		FDA-approved for the treatment of anorexia associated with weight loss in adult patients with AIDS
Testosterone [13, 66]	Testosterone	Transdermal gel, intramuscular injection, oral [13, 66]		No specifically approved indication for the treatment of HIVAW or HAWL
Anabolic steroids [13, 63, 69]	Nandrolone decanoate	Injection		No specifically approved indication for the treatment of HIVAW or HAWL No longer marketed or available in the US
	Oxandrolone	Oral		No longer FDA approved or available in the United States
Recombinant human growth hormone [58, 62]	Somatropin [57]	Subcutaneous injection; dose is weight based and begins at 0.1 mg/kg		FDA-approved for the treatment of PWH with HIVAW or HAWL to increase lean body mass and body weight, and improve physical endurance
Cytokine production [27, 65, 73] modulators	Thalidomide	Oral		No specifically approved indication for the treatment of HIVAW or HAWL

Nonpharmacological interventions	Improvement of LBM?	Principal routes of administration	Improvement with functional status?	Additional considerations
Nutritional supplements [61, 67, 68, 70, 72]	No; exercise required	Oral	No	Supplements not FDA approved for use May require dietary counseling
Resistance training [64, 71, 72]	Yes	N/A	Yes	May require access to equipment or facility

Abbreviations: FDA, Food and Drug Administration; HAWL, HIV-associated wasting; HIVAW, HIV-associated wasting; LBM, lean body mass; N/A, not applicable; PWH, people with HIV.

#### Appetite Stimulants

##### Dronabinol

Dronabinol, is an orally active cannabinoid that has complex effects on the central nervous system and is indicated for the treatment of anorexia associated with weight loss in PWH as 2.5-mg, 5-mg, and 10-mg oral capsules [74]. Among patients with AIDS-related anorexia for whom poor appetite contributes to weight loss, dronabinol demonstrated increased appetite, with observed trends towards improved body weight, improved mood, and decreased nausea [74]. Reported common adverse reactions in clinical trials (>1% incidence) of dronabinol included asthenia, cardiac palpitations, tachycardia, vasodilation/facial flush, abdominal pain, nausea, vomiting, dizziness, euphoria, paranoid reaction, somnolence, abnormal thinking, amnesia, anxiety/nervousness, ataxia, confusion, depersonalization, and hallucination [74]. Dronabinol does not have an FDA-approved indication for the treatment of HIVAW/HAWL.

##### Megestrol Acetate

Megestrol acetate (MA) is a synthetic derivative of progesterone reported to stimulate appetite and is indicated for the treatment of unexplained significant weight loss with a diagnosis of AIDS [75]. At the recommended dose, MA can result in increased LBM. Treatment with 800 mg of MA daily demonstrated significant improvement in appetite and significantly greater weight compared with placebo after 12 weeks [75]. Potential adverse events include thrombophlebitis, deep vein thrombosis, pulmonary embolism, and glucose intolerance [75].

#### Anabolic Steroids

##### Testosterone and Synthetic Derivatives of Testosterone

Anabolic steroids, including injectable testosterone, injectable synthetic derivatives of testosterone (eg, nandrolone) [76], and orally administered synthetic derivatives of testosterone (eg, oxandrolone) [58], have had various indications and applications for weight gain. However, these agents have not had or had not received FDA approval for the treatment of unexplained weight loss associated with HIVAW or HAWL [77]. However, these agents have not had or had not received FDA approval for the treatment of unexplained weight loss associated with HIVAW or HAWL [77]. Patients with hypogonadism should be screened and treated with recommended therapies such as testosterone [78]. In the absence of hypogonadism, testosterone is not formally indicated for the treatment of HIVAW or HAWL [77]. Although nandrolone is still FDA approved to treat certain medical conditions, it is no longer manufactured or marketed in the United States [79], and the FDA withdrew its approval of oxandrolone in June 2023 [80].

#### Recombinant Human Growth Hormone

Somatropin (Serostim®) is an injectable recombinant human growth hormone, which is the only FDA-approved treatment specifically for HIVAW or cachexia in PWH and is the only one in its class with FDA approval specifically for this indication [57]. Published data from clinical trials have demonstrated that the use of a 12-week course of Serostim® can significantly improve LBM, weight, and physical endurance compared with placebo [57]. Adverse events include development of edema, arthralgias, myalgias, and increases in blood glucose levels, which are often dose dependent and reversible on dose reduction or discontinuation [57]. An extension study of Serostim®, in which patients completing the initial 12-week placebo-controlled phase were randomly assigned to continue Serostim® treatment for up to 48 weeks, was performed [81]. The pattern of adverse events during the extension phase was similar to that observed during the initial 12-week phase. The safety and efficacy of Serostim® past 48 weeks, or the optimal length of use, have not been fully investigated. Healthcare professionals and their patients should take this into consideration when determining treatment course.

#### Cytokine Production Modulators

Inflammatory cytokines have been implicated in AIDS-related wasting [27]. It has been hypothesized that increased production of TNF-α can result in hypermetabolism and increased energy usage, leading to wasting [73]. Thalidomide, an inhibitor of TNF-α, was investigated as a potential treatment for HIVAW in the pre-ART era but is not an approved treatment for this indication.

### Nonpharmacological Treatments

#### Nutritional Supplements

Optimal nutrition is important for long-term health and the preservation of BCM, physical and cognitive function, and reduction of the risk of unintentional weight loss [72]. There are no specific dietary supplements indicated for the treatment or prevention of HIVAW or HAWL, and most nutritional supplements have not been reviewed or approved by the FDA. The use of some nutritional supplements (eg, arginine, Ω-3 fatty acids) can induce weight gain [67, 68]. PWH may benefit from increasing dietary protein intake to proactively prevent muscle wasting [82].

Patients receiving supplementation with whey protein have not experienced significant increases in weight or LBM [70]. There is evidence of improvement in overall body composition when nutritional supplements are combined with dietary counseling [61].

#### Physical Activity and Resistance Training

Physical activity has immense beneficial outcomes, including cardiovascular, bone strength, and mental health improvements [72]. Moderate-intensity exercise can increase endurance, functional capacity, and muscular strength among PWH [64]. Resistance training can safely increase strength and counter the effects of sarcopenia in an older population [71]. In an older population, exercises that help improve gait speed or grip strength would be most beneficial for improving functional status [42]. Systematic exercise involving strength training can increase LBM and improve cardiovascular fitness but has not been associated with weight gain per se.

### Treatment Goals

Clear treatment goals for persons with HIVAW or HAWL may help improve the ability of PWH to manage their care and set expectations. No guidelines currently exist that identify a magnitude of weight gain that constitutes an adequate response to a therapeutic intervention. A valid, objective evaluation of body weight includes a standard weight measurement obtained on a calibrated scale in a clinical setting. Although in-clinic weight measurement is most desirable for accuracy and reliability, home weight measurement may be acceptable (especially in the era of telemedicine and wearable health devices). The use of circumferential anatomical measurements, such as measuring mid-arm or mid-thigh circumference, may support a diagnosis of muscle mass loss; however, these measurements are rarely obtained in clinical settings and require measurer training and standardization. Easily reproducible measures of successful intervention for HIVAW and HAWL include serial weight and BMI; these can be supported by measures of physical functioning such as timed walk and chair rise (measures validated in older populations of PWH [42] but not specifically in the context of HIVAW or HAWL).

Members of a healthcare team with complementary expertise generally share medical decision-making to customize treatment goals that are reasonable and attainable for persons with HIVAW or HAWL. The precise duration of each intervention for optimal outcomes is unclear, but generally a documented increase in weight that approaches baseline or premorbid weight and/or improvement from an underweight BMI to a normal BMI is usually a goal of treatment, as is maintenance of weight after discontinuation of a course of treatment.

The goal of any therapy or treatment plan for HIVAW or HAWL should include the maintenance or improvement of physical resiliency, which can offer protective health benefits. Sustaining a healthy weight is imperative for maintaining optimal health outcomes and serves as a buffer against health challenges that can impact physical functioning and mental health [83]. Fostering and maintaining physical resilience has been shown to improve adherence to ART, which, in turn, can reduce the risk of HIVAW [84]. Likewise, interventions aimed at mitigating stigma, depression, and adverse social determinants of health may help to build resilience.

### Recommendations

- Pharmacological treatment options for managing unintentional weight loss in PWH should target the etiology of the weight loss and prioritize those interventions that induce weight gain comprised primarily of muscle (LBM) gain over those that exclusively or primarily promote fat gain
- The only currently available FDA-approved treatment option for unintentional weight loss in PWH is the recombinant human growth hormone Serostim® (somatropin). The efficacy and safety of Serostim® for longer than 48 weeks or the optimal duration of Serostim® treatment have not been investigated. Healthcare professionals and their patients should consider this when determining treatment with Serostim®
- Nonpharmacological treatment options (nutritional supplementation and resistance exercise) should always accompany pharmacological treatment options when available and feasible
- The expert panel recommends that PWH who unintentionally lose weight after initial successful weight gain efforts with pharmacologic therapies should undergo reevaluation. Consider reinitiation of the previously successful treatment option if determined to be medically appropriate for the patient
- Further clinical studies are necessary to evaluate preventive and treatment modalities in PWH who have or are at risk of HAWL.

## CONCLUSIONS

Since the introduction of virally suppressive combination ART, the incidence and prevalence of HIVAW has decreased significantly. However, recent estimates regarding the prevalence (12%) and incidence (7%) of unintentional weight loss among PWH receiving ART (particularly those aged >50 years) provide evidence that this clinical entity still exists [9]. An expert panel described a more contemporary definition of involuntary weight loss among PWH, which we reference here as HAWL, occurring among PWH receiving effective ART with virologic suppression, often older in age, and experiencing unintentional weight loss nevertheless. Long-term treatment goals for these patients should include achieving weight stability as a critical step in maintaining overall health, optimal functional status, and resilience. These measures, in turn, are likely to maximize healthy life spans and foster long-term independence. As such, our proposed definition of HAWL was developed to raise awareness of this treatable condition and to aid clinicians in accurate and timely diagnosis.

Rapid recognition and accurate diagnosis of HAWL and the provision of targeted effective treatment while providing appropriate care are critical for reducing morbidity, mortality, and maintenance of long-term health. Although there are no sensitive or specific routinely available tests or biomarkers to confirm a diagnosis of HAWL, clinicians should be cognizant of the utility of serial weight measurements for documenting unintentional weight loss among at-risk patients. Patient-reported measures can be used as an important method to complement and supplement objective measures of weight and functioning, and their clinical use may contribute to a sense of empowerment and self-advocacy among PWH.

Much can be learned about aging from research with validated instruments used routinely in geriatric medicine. These can be applied to the diagnosis, monitoring, and treatment of PWH at risk for or with HAWL. Various treatment modalities have been explored in the management of HAWL, but few head-to-head comparisons of interventions or long-term data to inform their best use exist. Treatment approaches for HAWL will require individualization, but an agreed-on modern diagnostic approach is an important first step in reducing morbidity and mortality while improving healthy life spans of PWH.

**Please see the Indication and Important Safety Information about the EMD Serono product mentioned in this paper below and its full Package Insert enclosed.**

## INDICATIONS AND USAGE

Serostim® (somatropin) for injection is indicated for the treatment of those with HIV with wasting or cachexia to increase LBM and body weight and improve physical endurance. Concomitant antiretroviral therapy is necessary.

## IMPORTANT SAFETY INFORMATION

### Contraindications

**Acute Critical Illness:** Serostim® should not be initiated in patients with acute critical illness resulting from complications following open heart or abdominal surgery, multiple accidental trauma, or acute respiratory failure.

**Active Malignancy:** Somatropin is contraindicated in the presence of active malignancy. Any preexisting malignancy should be inactive and its treatment complete before instituting therapy with somatropin. Discontinue somatropin if there is evidence of recurrent activity.

**Hypersensitivity:** Serostim® is contraindicated in patients with a known hypersensitivity to somatropin or any of its excipients. Systemic hypersensitivity reactions have been reported.

**Diabetic Retinopathy:** Somatropin is contraindicated in patients with active proliferative or severe nonproliferative diabetic retinopathy.

### WARNINGS and PRECAUTIONS

**Acute Critical Illness:** Increased mortality (42% vs 19% in somatropin compared to placebo treated) in patients with acute critical illness due to complications following open heart surgery, abdominal surgery or multiple accidental trauma, or those with acute respiratory failure has been reported after treatment with pharmacologic amounts of somatropin.

**Concomitant Antiretroviral Therapy:** Somatropin has been shown to potentiate HIV replication in vitro, and there was no increase in virus production when antiretroviral agents were added to the culture medium. No significant somatropin-associated increase in viral burden was observed. All patients received antiretroviral therapy for the duration of treatment during Serostim® clinical trials.

**Neoplasms:** Patients with preexisting tumors should be monitored for progression or reoccurrence. Monitor patients on somatropin therapy carefully for preexisting nevi.

**Impaired Glucose Tolerance/Diabetes:** Patients with other risk factors for glucose intolerance should be monitored closely during Serostim® therapy. Cases of new-onset impaired glucose tolerance, new-onset type 2 diabetes, and exacerbation of preexisting diabetes have been reported in patients receiving Serostim®. Some patients developed diabetic ketoacidosis and diabetic coma and, in some, improved when Serostim® was discontinued and in others persisted. Some of these patients required initiation or adjustment of antidiabetic treatment.

**Intracranial Hypertension:** Intracranial hypertension with papilledema, visual changes, headache, nausea, and/or vomiting has been reported usually within the first 8 weeks of somatropin therapy and rapidly resolved after stopping or reducing the somatropin dose. Funduscopic examination should be performed before initiating treatment with somatropin and periodically during treatment. If papilledema is observed, treatment should be stopped and restarted at a lower dose after intracranial hypertension–associated symptoms have resolved.

**Severe Hypersensitivity:** Serious systemic hypersensitivity reactions including anaphylactic reactions and angioedema have been reported with postmarketing use of somatropin products. Patients and caregivers should be informed that such reactions are possible and that prompt medical attention should be sought if an allergic reaction occurs.

**Fluid Retention/Carpal Tunnel Syndrome:** Increased tissue turgor (swelling, particularly in the hands and feet) and musculoskeletal discomfort (pain, swelling and/or stiffness) may occur during treatment with Serostim®, but may resolve spontaneously, with analgesic therapy, or after reducing the frequency of dosing. Carpal tunnel syndrome may occur and if the symptoms of carpal tunnel do not resolve by decreasing the weekly number of doses, it is recommended that Serostim® treatment be discontinued.

**Skin Atrophy:** Rotate the injection site to avoid tissue atrophy.

**Pancreatitis:** Cases of pancreatitis have been reported rarely. Consider pancreatitis in patients who develop persistent severe abdominal pain.

### ADVERSE REACTIONS

In clinical trials in HIV-associated wasting or cachexia the most common adverse reactions (incidence >5%) were arthralgia, myalgia, peripheral edema, arthrosis, nausea, paresthesia, generalized edema, gynecomastia, hypoesthesia, and fatigue.

**SPECIAL POPULATIONS:** Somatropin should be used during pregnancy only if clearly needed and with caution in nursing mothers because it is not known whether somatropin is excreted in human milk. The safety and effectiveness of somatropin in pediatric patients with HIV have not been established. Clinical studies did not include sufficient numbers of subjects aged ≥65 years to determine a response different from that of younger patients. Studies have not been conducted in patients with hepatic or renal impairment. Gender-based analysis is not available.

All trademarks are the property of their respective owners. SEROSTIM® is a trademark of Merck KGaA, Darmstadt, Germany, or its affiliates. EMD Serono is the pharmaceutical business of Merck KGaA, Darmstadt, Germany, in the United States and Canada.

## Notes

***Financial support*.** This work was sponsored by EMD Serono, Inc.

***Supplement sponsorship*.** This article appears as part of the supplement “Expert Consensus Statement on an Updated Definition of Unintended Weight Loss Among Persons With HIV in the Modern Treatment Era,” sponsored by EMD Serono.
